# The LEGSKO Mouse: A Mouse Model of Age-Related Nuclear Cataract Based on Genetic Suppression of Lens Glutathione Synthesis

**DOI:** 10.1371/journal.pone.0050832

**Published:** 2012-11-30

**Authors:** Xingjun Fan, Xiaoqin Liu, Shuyu Hao, Benlian Wang, Michael L. Robinson, Vincent M. Monnier

**Affiliations:** 1 Department of Pathology, Case Western Reserve University, Cleveland, Ohio, United States of America; 2 Case Center for Proteomics and Mass Spectrometry, Case Western Reserve University, Cleveland, Ohio, United States of America; 3 Department of Zoology, Miami University, Oxford, Ohio, United States of America; 4 Department of Biochemistry, Case Western Reserve University, Cleveland, Ohio, United States of America; University of Missouri-Columbia, United States of America

## Abstract

Age-related nuclear cataracts are associated with progressive post-synthetic modifications of crystallins from various physical chemical and metabolic insults, of which oxidative stress is a major factor. The latter is normally suppressed by high concentrations of glutathione (GSH), which however are very low in the nucleus of the old lens. Here we generated a mouse model of oxidant stress by knocking out glutathione synthesis in the mouse in the hope of recapitulating some of the changes observed in human age-related nuclear cataract (ARNC). A floxed Gclc mouse was generated and crossed with a transgenic mouse expressing Cre in the lens to generate the LEGSKO mouse in which *de novo* GSH synthesis was completely abolished in the lens. Lens GSH levels were reduced up to 60% in homozygous LEGSKO mice, and a decreasing GSH gradient was noticed from cortical to nuclear region at 4 months of age. Oxidation of crystallin methionine and sulfhydryls into sulfoxides was dramatically increased, but methylglyoxal hydroimidazolones levels that are GSH/glyoxalase dependent were surprisingly normal. Homozygous LEGSKO mice developed nuclear opacities starting at 4 months that progressed into severe nuclear cataract by 9 months. We conclude that the LEGSKO mouse lens mimics several features of human ARNC and is thus expected to be a useful model for the development of anti-cataract agents.

## Introduction

Cataract is a leading cause of blindness, accounting for 50% of blindness worldwide [1]. The cumulative incidence of cataract is strongly age-related and ranges from 2% at ages 45–54 years to 45% at ages 75–85 [1], with nuclear cataracts accounting for 30% of all age-related cataracts [2]. Surgical removal of the cataractous lens remains the only therapy, yet the National Eye Institute has estimated that a ten-year delay in the onset of cataract would result in a 50% reduction in the prevalence of cataract [3]. Both lens nuclear opacity and nuclear cataract surgery are associated with increased mortality according to the Beaver Dam Eye Study [1] and the Age-Related Eye Disease Study (AREDS) [4]. Thus, understanding the pathogenesis of age-related nuclear cataracts remains an important goal of vision research that may also provide clues on broader mechanisms of aging.

Age-related cataract is strongly related to the accumulation of damage to its long-lived proteins, the crystallins. Major age-related lens protein modifications include deamidation, deamination, racemization, accumulation of truncation products, accumulation of UV active, fluorescent, and non-UV active protein adducts and crosslinks from glycation, ascorbylation and lipoxidation reactions [5]. Collectively, these modifications contribute toward decreasing protein stability, partly by impairing the chaperone function of α-crystallins, the levels of which decrease with age due to insolubilization [6]. Overall, the aging human lens is constantly exposed to chemical and physical stresses. However, while oxidative damage is subdued during normal aging, it is a major cause or consequence of nuclear cataracts, the most common types of age-related cataracts, whereby the loss of glutathione (GSH) and formation of disulfides are considered to be the key factors in oxidative stress and nuclear cataractogenesis [7].

To protect from oxidation the lens has evolved as an anaerobic system with millimolar concentrations of both glutathione (GSH) and ascorbic acid. However, both protective systems are impaired during aging whereby GSH level significantly declines in the lens nucleus [8], [9]. This is in part attributed to lowered γ-glutamyl-cysteine ligase (Gcl) activity [10] and a barrier to GSH diffusion toward the nucleus [9]. As a result ascorbic acid is increasingly oxidized throughout life leading to accelerated accumulation of crystallin-bound advanced glycation end products (AGEs) that contribute to cataractogenesis [11], [12], [13]. Concomitantly, increased protein residue oxidation is observed, as reflected by the formation of methionine sulfoxide, protein disulfides, kynurenine, and o-tyrosine from methionine, cysteine, tryptophan and phenylalanine, respectively [13], [14], [15].

In spite of considerable progress in the field, it has been extraordinarily difficult to study the relationship between the protein modifications and carbonyl stress or oxidant stress due to lack of appropriate animal models. One recent model of carbonyl stress developed by us successfully mimics the carbonyl stress component of the aging lens [12]. However, while several models illustrate the role of glutathione for sulfhydryl homeostasis, its role for lens transparency during aging has not been unequivocally established. Indeed most chemically or genetic induced models of disrupted GSH homeostasis only produced opacity in pups or very young animals, with uncertainties as to whether the observed lenticular changes were due to developmental abnormalities or chemical toxicity via pathways unrelated to oxidation itself. For this reason, we set out to genetically lower lenticular glutathione levels specifically in the lens (since the systemic knockout is lethal [16]) by disrupting the catalytic subunit of γ-glutamyl- cysteine ligase (Gclc) using a conditional Cre/LoxP approach. The predicted slow decline in glutathione levels using this approach is hypothesized to mimic the processes underlying the oxidative arm of human ARNC. Below we present the genetic, biochemical and biological phenotypes of resulting from the loss of Gclc function in the lens of the Lens Glutathione Synthesis KnockOut (LEGSKO) mouse.

## Results

### Conditional Deletion of Gclc Impairs Lens GSH Synthesis

In order to specifically delete Gclc from the lens, we crossed the Gclc^fl/fl^ mice with MRL10-Cre mice [17] that express Cre recombinase in lens epithelia and fibers to ultimately generate mice homozygous for the conditional allele and hemizygous for MLR10 transgene. These mice Gclc^−/−/^MRL-10^+/−^ are deficient for Gclc specifically in the lens and are, herein named homozygous lens GSH knockout mice (HOM-LEGSKO). Similarly, the Gclc^fl/+^/MRL-10^+/−^ mice were named heterozygous lens GSH knockout mice (HET-LEGSKO) and should exhibit reduced Gclc levels in the lens. No lens abnormalities have been reported for mice that are hemizygous or homozygous for the MRL10-cre transgene in the absence of floxed alleles [17], and therefore phenotypes manifested in LEGSKO mice were contributed by Gclc deficiency alone. The LEGSKO mice were continuously crossbred with Gclc^fl/fl^ mice (C57BL/6) to convert the genomic background towards C57BL/6. All the data provided in this paper are based on B6/FVB mixed background at third generation bred mice. The same breeding pattern and age matched control mice were used as wild type controls (Gclc^fl/fl^).

**Figure 1:**
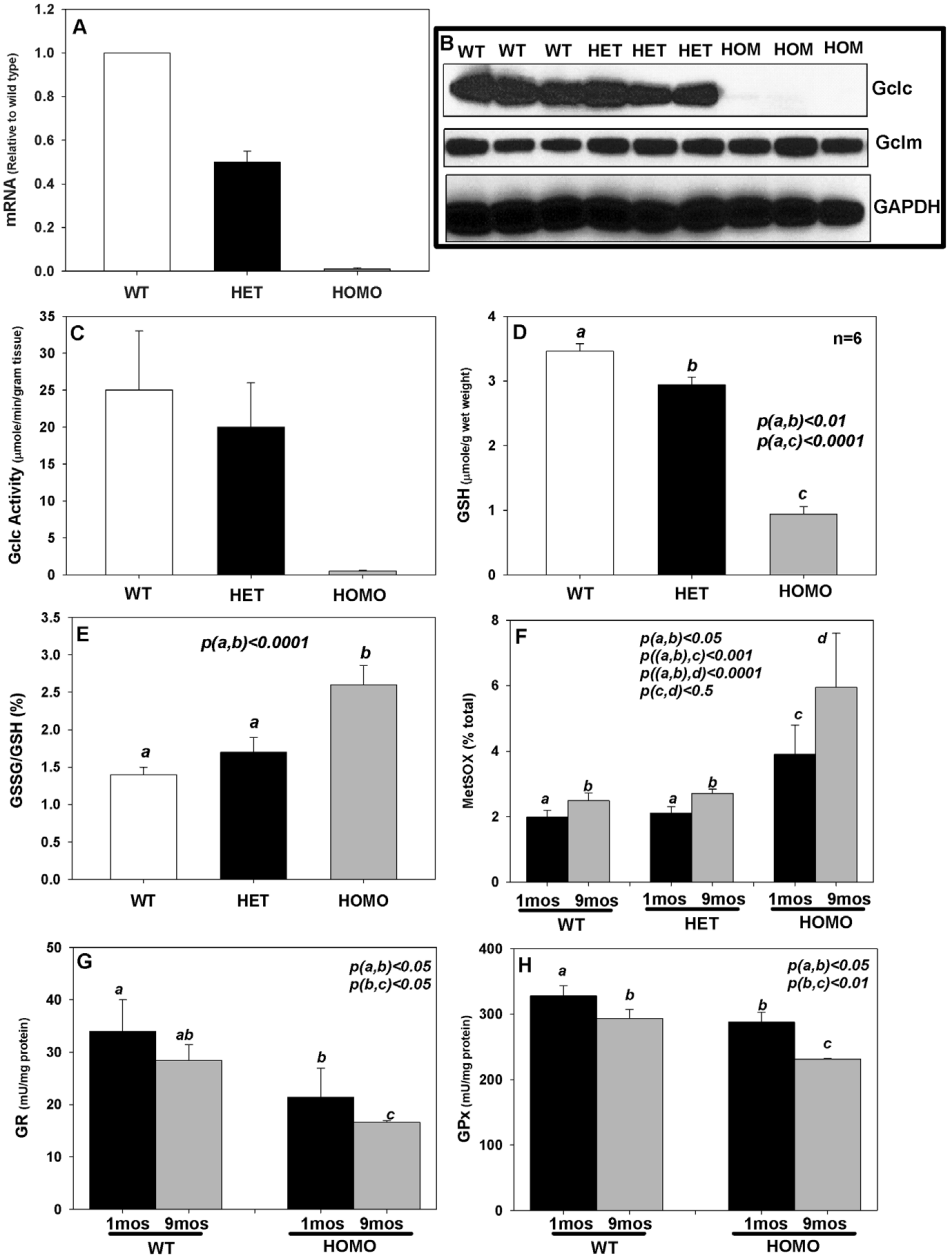
Charaterization of the GSH genotype and phenotype of LEGSKO mouse. 3mos old LEGSKO mouse lens (without cataract) total RNA was extracted and Gclc mRNA was determined by real-time PCR. The data was calculated relative to age matched wild type lenses. Gclc enzyme activity was determined by HPLC by monobromobimane fluorescent derivatization. Gclc protein expression was determined by Western-blot. (A). Gclc mRNA was reduced 50% in heterozygous mouse lenses and completely abolished in homozygous mouse lenses (n = 6). (B). Gclc protein level was reduced in heterozygous mouse lenses and abolished in homozygous lenses. Mouse liver protein extract was used as positive control. (C). Gclc activity was mildly reduced in heterozygous mouse lenses and quasi no detectable in homozygous mouse lenses (n = 6). (D). Fresh lenses (n = 6) were snap frozen, thaw at room temperature for 1 min and dissected immediately. The protein was precipitated, and the supernatant was subjected to GSH analysis by glutathione reductase (GR) and β-NADPH enzymatic recycling method. (E). the percentage of GSSG vs. GSH was significantly (p<0.0001) elevated in homozygous mouse lenses vs. wild type/heterozygous lenses. (F). Methionine sulfoxide was significantly (p<0.001) elevated in homozygous vs. wild type/heterozygous lenses, and also significantly increased with age (P<0.05). (G). Glutathione reductase (GR) activity, expressed as unit per milligram of protein, was mildly but significantly (P<0.01) reduced in homozygous lenses vs. wild type, and also significantly reduced with age. (H). Glutathione peroxidase (GPx) activity, expressed as unit per milligram of protein was mildly but significantly reduced in homozygous lens at 9 months old of age (p<0.05). One-Way ANOVA followed by Post-Hoc analysis was used for all comparisons (n = 10, per group, except GSSG/GSH which is 6 per group).

LEGSKO mice exhibited reduced expression of Gclc transcripts and protein. HOM-LEGSKO lenses exhibited nearly undetectable levels of Gclc mRNA by real-time PCR, and Gclc transcripts were reduced nearly 50% in HET-LEGSKO lenses compared to wild type lenses (Fig. 1A). The levels of Gclc mRNA and protein were indistinguishable between Gclc^fl/fl^ lenses (without MLR10 transgene) and lenses of wild-type mice (data not shown). The lens Gclc protein expression was completely abolished in HOM-LEGSKO lenses compared to wild type lenses based on western-blot analysis (Fig.1B). This was also confirmed by immunohistochemistry analysis using monoclonal Gclc antibody (data not shown). The deletion of Gclc gene had no impact on glutamate-cysteine ligase, modifier subunit (Gclm) protein level (Fig. 1B). Most importantly, the Gclc activity determined by monobromobimane derivatization and HPLC analysis with fluorescence detection clearly demonstrated no detectable activity in HOM-LEGSKO lenses (Fig.1C). Interestingly, however, there was only 20% reduction of Gclc activity in HET-LEGSKO lenses compared to wild type lenses. HET-LEGSKO lenses had a ∼50% reduction of Gclc mRNA (Fig.1A) and 25% lower protein expression (Fig. 1B). The most intriguing finding was that HET-LEGSKO mice lenses maintained quasi-normal GSH level (reduced <10%), while HOM-LEGSKO GSH levels were more than 60% reduced compared to wild type lenses at 3months of age (Fig.1D). These results indicate that compensatory mechanisms might be involved in lens GSH homeostasis, most likely via transporter(s) systems as suggested by others [18]. Moreover, analysis of cortical and nuclear GSH content in the HOM-LEGSKO lenses at 5 months of age (Table 1) revealed a GSH gradient from cortex to nucleus, with over 75% decreased in GSH in the nuclear vs. only a 50% decrease of GSH within the cortical region.

**Table 1:** GSH and GSSG level (n = 6).

Genotype	Age (wks)	GSH (µmole/g wet weight)		GSSG(µmole/g wet weight)	
		Cortical	Nucleus	Cortical	Nucleus
WT	4	3.23±0.23	3.07±0.21	0.031±0.005	0.03±0.006
LEGSKO	4	1.62±0.18	1.46±0.10	0.027±0.004	0.022±0.003
WT	36	3.08±0.33	2.65±0.39	0.03±0.007	0.032±0.003
LEGSKO	36	0.97±0.29	0.47±0.10	0.22±0.003	0.11±0.001
LEGSKO Cataract	36	0.89±0.31	N.D	0.21±0.005	N.D

N.D. : not detectable.

### Impact of Suppressed GSH Levels on the Lens: Elevated ROS

**Figure 2:**
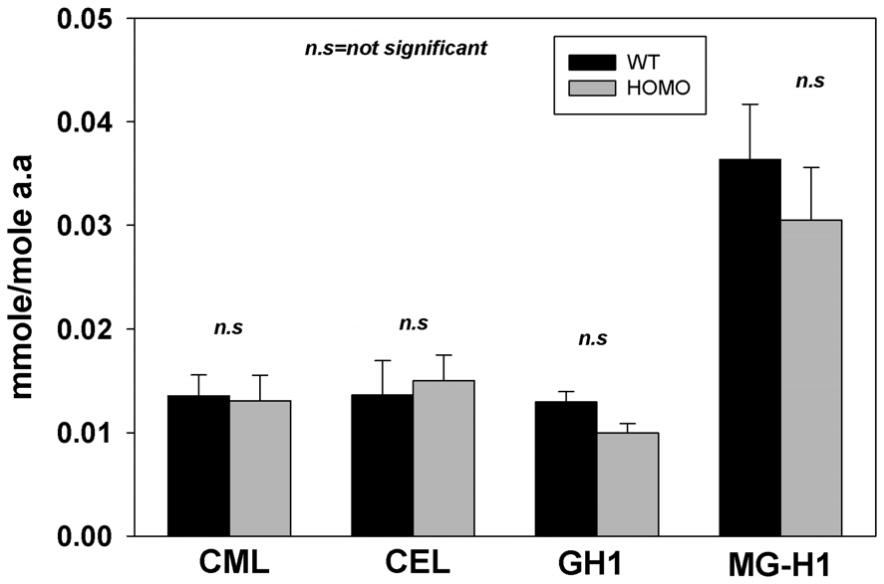
Quantitative comparison of mean levels ± SD for protein modification by glycation at 9 mos old of WT and Homo-LEGSKO mice lenses. Carboxymethyl-lysine (CML), carboxyethyl-lysine (CEL), methylglyoxal hydroimidazolone-1 (MG-H1), and glyoxal hydroimidazolone-1 (G-H1) were determined by LC/MS. No significant (n.s) change was observed between WT and LEGSKO lenses. Student’s *t* test was used for data analysis.

The GSSG/GSH ratio, an index of ROS production, was mildly and strongly increased in HET-LESGKO vs. HOM-LEGSKO mice, respectively (Fig. 1E). One of the major protein modifications associated with oxidative stress during human lens aging is methionine sulfoxide (MetSOX), which increases with age and is highly elevated in cataractous lenses, with over 50% of membrane bound protein methionine present in oxidized form [15], [19]. LC/MS based methionine sulfoxide determination indicated that the HOM-LEGSKO lenses protein bound methionine oxidation was significantly increased compared to heterozygous and wild type control lenses, and it was also elevated with age as measured at 9 months compared to 3 months of age (Fig. 1F). GSH antioxidative cycle enzymes glutathione reductase (GR) and glutathione peroxidase (GPx) activity were mildly but significantly decreased (Fig. 1G&F). Surprisingly, however, we did not find elevation of protein modification by glycation in HOM-LEGSKO mice (Fig.2), such as carboxymethyl-lysine (CML), carboxyethyl-lysine (CEL), methylglyoxal hydroimidazolone-1 (MG-H1), and glyoxal hydroimidazolone-1 (G-H1), which are known to positively correlate with aging in the human lens [20], [21]. An explanation for this finding is provided in the Discussion.

**Figure 3:**
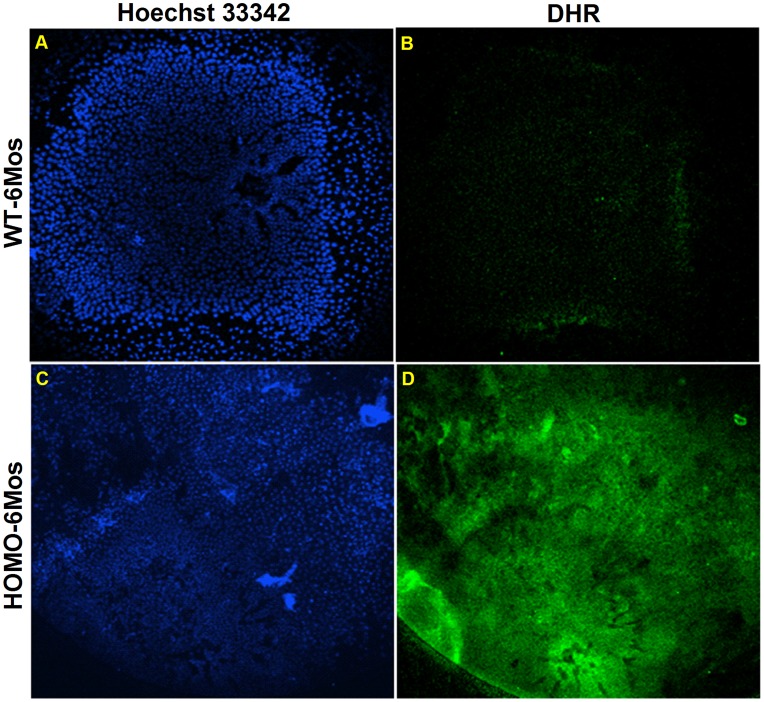
Comparison of lens anterior surfaces of 6 mos wild type and Homo-LEGSKO mouse lenses vitally stained for DNA and ROS. Fresh lenses were stained with both Hoechst 33342 and Dihydrorhodamine 123 (DHR). The Lens anterior was scanned using 10X objective, and 65 µm deep into the cortex and 0.8 µm layer Z-scan was performed and projection image was obtained with same method for both WT and LEGSKO lenses. (A) and (C). Comparison of DNA staining with Hoechst 33342 (blue) of the anterior surface of Wt and LEGSKO lenses. (B) and (D). Comparison of vital staining for ROS fluorescence with DHR stain (green) of the same surface areas.

As the 40% reduction of GSH in the cortical region was predicted to increase oxidative stress, we investigated ROS production in living lenses ex vivo. Freshly isolated 6 months old HOM-LEGSKO and age matched control lenses were stained vitally with dihydrorhodamine 123 (DHR), a reactive oxygen species marker, and co-stained DNA with Hoechst 33342 to mark lens cell nuclei. Fluorescence (green) of DHR indicated much stronger ROS also present at cortical region of HOM-LEGSKO lens vs. age matched control lens (Fig.3).

### Impact of Suppressed GSH Levels on Lens Transparency

**Figure 4:**
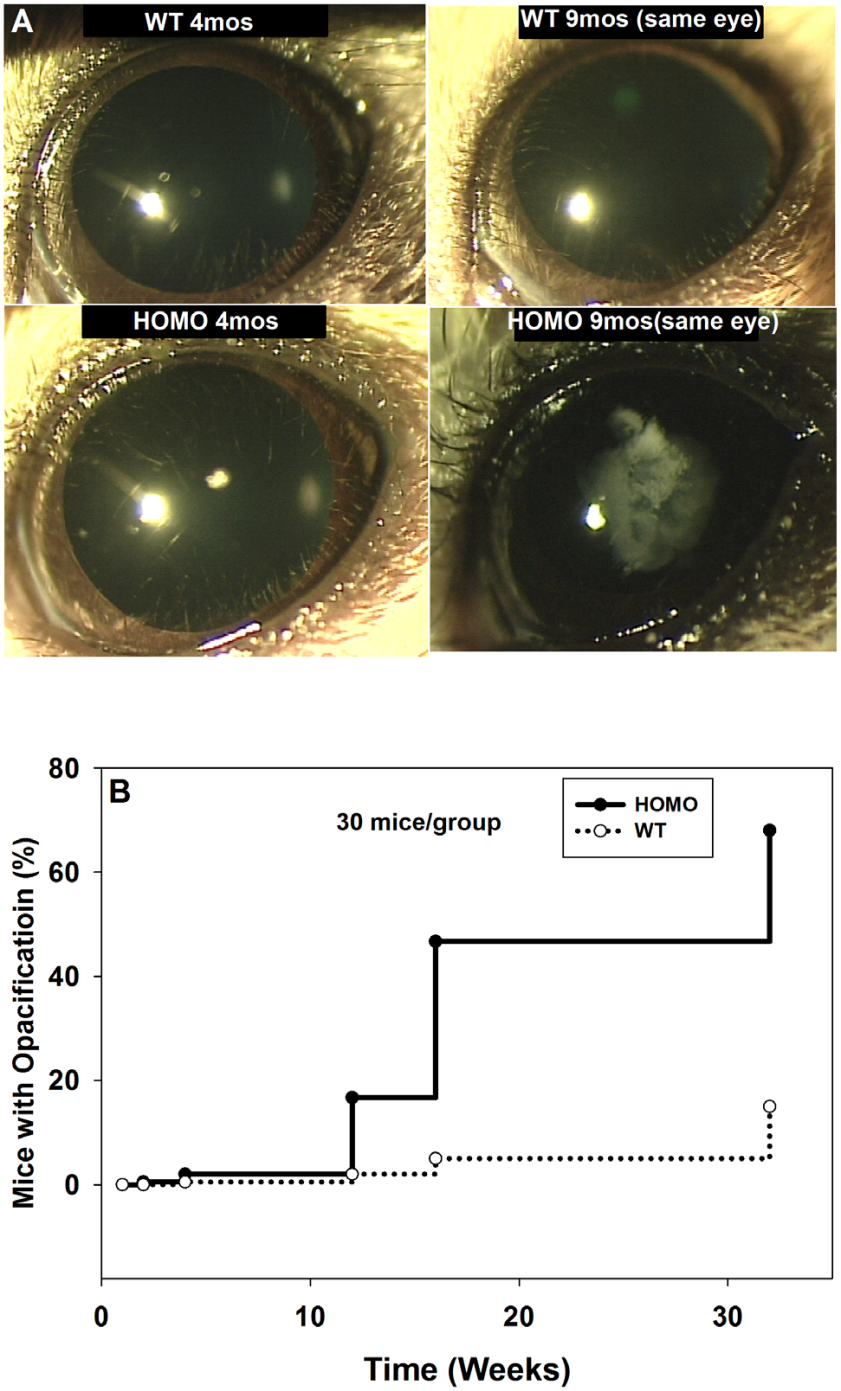
Slit-lamp image of LEGSKO and age matched wild type mouse lens. (A). Representative images of HOM-LEGSKO mouse lens at 4 and 9 months compared with age matched control wild type mouse lens. The same mouse was followed over a period of 9 months and lens images were taken every two months. (B). Slit-lamp images were taken periodically and lens opacity/cataract of any size were recorded for 9 months in homozygous LEGSKO mice vs. age matched wild type mice (n = 30 mice per group). LEGSKO mice developed lens opacities and cataract at much higher rate compared to wild type mice.

About 20% of the homozygous mice developed nuclear opacification starting at 3 months of age based on the sensitivity of Slit-lamp detection, which progressed into severe nuclear cataract at 9 months age. In this report, we define opacity as a white area the size of at least 0.3 micrometer diameter. A typical cataract image (Fig. 4A) shows the same lens at 4 and 9 months of age, whereby the nuclear cataract was found to progress from a small opacity at 4 months to severe nuclear cataract at 9 months. The LEGSKO lenses have relative similar size and weight compared to wild type lenses. The GSH level in the lens nucleus was barely detectable and GSH level in the lens cortex dropped over 60% compared to age matched wild type lenses (Table 1).

The kinetics of the cataract formation was assessed quantitatively using Slit-lamp (Fig. 4B). 50% of HOM-LEGSKO mice had mature cataracts by 4 months compared to age matched wild type mice.

## Discussion

This study shows that genetic suppression of lenticular glutathione synthesis generates phenotypic protein and GSH homeostatic changes that closely resemble those present in the nuclear region of the aging human lens, including cataractogenesis. Several authors have previously contributed to this paradigm by demonstrating that e.g. gamma-glutamyl transpeptidase deficient mice develop nuclear opacities at 1 week secondary to cysteine deficiency [22], or that treatment with buthionine sulfoxide (BSO) results in lowered GSH levels and cataract in newborn rats [23] and very young mice [24] ^.^ Vice-versa, oxidative stress from hyperbaric oxygen duplicates many changes observed in the aging human lens [25]. Knocking out glutathione peroxidase (GPX-1) in mice leads to cataract formation [26], whereby changes in lens epithelial cell morphology and DNA breaks were also found under oxidant stress generated by either hydrogen peroxide (H_2_O_2_) treatment or GPX-1 null condition [27]. Lens cells behave similarly when glutathione reductase or glutathioredoxin 2 synthesis have been knocked out [28], [29]. Interestingly, in mouse lenses, free GSH detoxifies more H_2_O_2_ (54% to 72%) than GPX-1 (15%) or catalase (30%) [30].

In old or cataractous human lens there is a GSH gradient from the cortical to the nuclear region [8]. Truscott et al [9] proposed that a diffusion barrier forms with age, and therefore is an impediment for GSH diffusion. The data from LEGSKO mouse lenses confirm the presence of this gradient with non-detectable GSH in the nucleus of cataractous lenses. These data suggest that a barrier may also form in mouse lenses, and that impaired GSH synthesis is in part responsible for the gradient. Takemoto et al [31] discovered that crystallins were involved in inter-disulfide cross-linking with membrane, and tight binding of protein to the membrane has been proposed as a potential mechanisms of barrier formation in aged human lenses [32]. In human, lens nucleus enriched crystallins, such as gamma crystallins, have large numbers of free cysteine residues (i.e. 8 cys in human gamma C) compared to alpha crystallin, which only have two cysteine residues. These cysteine residues are maintained in either free form or as intramolecular disulfides to maintain crystallin protein stability in young and normal lenses. However, during aging, with elevated oxidation from reduced GSH level, these cysteine residues are oxidized and form intermolecular disulfide crosslinks. Such transition may impact on crystallin stability, and may cause crystallin unfolding and formation of aggregates with other proteins. Studies will be needed to elucidate the fundamental nature of the protein changes under reduced GSH levels. Because the LEGSKO mouse lenses are too young to accumulate other modifications than those resulting from oxidation, and because these mice are free of diabetes, it will be possible to selectively study the consequences of protein oxidation for protein aggregation.

It has been suggested that maintenance of a threshold of GSH concentration above 1 mM in human lens nucleus was critical for keeping the lens transparent [9], [33]. This may explain the rapid cataract rate of LEGSKO mice at 9mos, since nuclear GSH is in much lower level i.e. 0.5 mM). In addition, ascorbic acid oxidation may play a key role in crystallin damage in the human lens nucleus when the GSH level drops below a certain threshold. In contrast, the mouse lens has barely detectable ascorbic acid [12]. Furthermore, we found elevated oxidation in the cortical region of LEGSKO lenses compared to wild type lenses, but we did not observe opacity in cortex at 9mos of LEGSKO mice. Thus it remains to be seen if cortical cataract will also form with age.

Another important function of GSH is the detoxification of the oxoaldehydes glyoxal and methylglyoxal, the latter being one of the most important sources of protein damage in aging and cataractous lenses [12], [13], [34]. For this reason we expected to find increased levels of carboxymethyl-lysine (CML), carboxyethyl-lysine (CEL) and MG-H1 hydroimidazolone. At first we were surprised that none of these modifications were increased. This may mean there was enough residual glutathione as cofactor of glyoxylase I for the detoxification of these AGE precursors. However, the most likely explanation is that AGE levels are very low because vitamin C is absent in normal and LEGSKO mouse lens (both are ∼100 µM), except in the hSVCT2 transgenic mouse [12]. Indeed previous data from our and other laboratories have unequivocally established ascorbic acid as a major source of these AGEs in the aging lens [12], [35], [36]. Breeding experiments between the LEGSKO mouse and the hSVCT2 mouse will confirm the extent to which the GSH/glyoxalase system is important for protection against ascorbic acid derived AGEs. Similarly, exposure of the hSVCT2, LEGSKO and hSVCT2xLEGSKO F1 mouse to UV/VIS light is expected to provide important in vivo information on the role of GSH for protection against photoxidative damage and formation of photosensitizers resulting from tryptophan oxidation.

In summary, lens-specific suppression of GSH synthesis in the LEGSKO mouse, while maintaining all other metabolic body functions intact, resulted in biochemical, biological and cataractous changes mimicking those of age-related human nuclear cataracts. Thus, the LEGSKO mouse is expected to be a useful tool for the development of pharmacological agents that can either restore the antioxidant reserve of the lens or block the distal effects of oxidant stress resulting from GSH deficiency.

## Materials and Methods

### Creation of Gclc Floxed Mouse

Gclc conditional knockout (Gclc^fl/fl^) target ES cells with C57BL/6 genetic background were obtained from the European Conditional Knockout Mouse Program (EUCOMM). The Gclc^fl/fl^ exon 2 is flanked by two loxP sites, and the Neo cassette is flanked by two FRT sites. Microinjection of ES cells into C57BL/6J blastocysts was done at the University of Michigan Transgenic Facility, and resulting chimeric male mice were bred with albino female mice to obtain the germ-line. F1 heterozygous Gclc^fl/fl^ mice were bred with mice carrying a ubiquitously expressing Flp knockin mouse (the Jackson lab, stock No. 009086) on a C57BL/6 background to remove the Neo cassette. The resulting Gclc^fl/fl^ mice were cross-bred to generate the homozygous Gclc^fl/fl^ mouse line. The presence of a floxed Gclc allele was identified by PCR using tail DNA with a primer pair (forward primer, 5′-CATGAGGAA CTGAACTGAAGGATTGA-3′; reverse primer, 5′-CAAGGAGGCTCACACATCCCAGAAC-3′), which generated a 310-bp amplicon.

### Creation of Lens Conditional Knockout Mouse (Gclc^fl/fl^/MRL10^+/−^)

In order to delete Gclc specifically from the lens epithelium and fibers, we crossed the Gclc^fl/fl^ mice with MRL10-Cre mice (FVB background) that express Cre recombinase in lens epithelium and fibers [17]. F1 mice (*Gclc^fl/wt^/MRL10^+/−^)* were backcrossed to Gclc^fl/fl^ mice to generate homozygous lens conditional knockout mice (*Gclc^fl/fl^/MRL10^+/−^*). All the data provide in this paper is based on B6/FVB mixed background. Mice conditionally lacking *Gclc* in lens (*Gclc^fl/fl^/MRL10^+/−^*), hereafter called LEGSKO, were identified by PCR using both conditional allele (Gclc^fl/fl^) genotype primer pair and primers pair that amplifies a 190-bp fragment of Cre recombinase coding region (forward primer, 5′-AATTTGCCTGCATTACCGGTCGATGCAACG-3′ and reverse primer 5′-CCATTTCCGGTTATTCAACTTGCACCATGC-3′). Mice were housed under diurnal lighting conditions and allowed free access to food and water. All animals were used in accordance with the guidelines of the Association for Research in Vision and Ophthalmology for the Use of Animals in Ophthalmology and Vision Research, and experimental protocols for this study were approved by the Institutional Animal Care and Use Committee (IACUC) of Case Western Reserve University.

### Quantitative Determination of Reduced Glutathione (GSH) and Oxidized Glutathione (GSSG)

Eyes were removed immediately after sacrifice. Lenses were homogenized in 200 µl ice-cold 5% metaphosphoric acid and 0.6% sulfosalicylic acid mixture in 0.1 M potassium phosphate buffer and 5 mM EDTA buffer (pH 7.5). The supernatant was analyzed for GSH and GSSG using glutathione reductase (GR) and β-NADPH enzymatic recycle method followed by colorimetric assay after derivatization with 5,5′-dithio-bis(2-nitrobenzoic acid) (DTNB) as described [37]. Commercially available GSH (G6529, Sigma, St. Louis) and GSSG (49740, Sigma, St. Louis) were used for standard curve calibration.

Lens fibers were subdivided onto cortex and nuclear fraction by a freeze-thawing technique whereby quick freezing at −80°C was followed by thawing at room temperature for 1 min, creating a clear white opacity in nucleus. The cortical and nucleus regions can then be easy separated for GSH and GSSG determination.

### γ-Glutamyl Cysteine Ligase Activity Assay

Gcl activity in lens extract was measured using monobromobimane derivatization and HPLC analysis with fluorescence detection as previously reported [38].

### RNA Extraction and Quantitative mRNA Analysis of Gene Expression

Minimum Information for Publication of Quantitative Real-Time PCR Experiments (MIQE) guidelines [39] was followed for real time PCR analysis. Eyes were removed immediately after sacrifice. Lenses were collected and quickly frozen with liquid nitrogen and stored at −80°C. Total RNA was prepared from single lens using TRIzol reagent (Invitrogen, Grand Island, NY). RNA concentration was measured with a Nanodrop instrument (2000c, Thermal Sci). Only RNA samples with UV 260 nm/280 nm ratio of 1.9–2.0 and UV 260 nm/230 nm ratio >1.8 were used for subsequent experiments. For real time PCR, total RNA was deoxyribonuclease-treated (Invitrogen, Grand Island, NY) and transcribed to complementary DNA with oligo(dT) or random hexamer primer (Invitrogen, Grand Island, NY) and Moloney murine leukemia virus reverse transcriptase (New England Biolabs, Ipswich, MA ). Real time PCR was performed with the SYBR Green method and an Applied Biosystems instrument (ABI StepOne Plus). Relative expression was calculated using the ΔΔCt method with normalization to constitutive genes as indicated in the figure legends. Specific primer sequences are available on request. All reactions were performed in triplicate. Data analysis was carried out using the cycle threshold values of target gene expression normalized by GAPDH as the internal control.

### Western Blot

Lens fiber fractions (cortex or nucleus) were collected in 50 mM potassium phosphate buffer (pH 7.4), homogenized, and centrifuged. Whole protein extracts were further processed for immunoblot analysis and probed against Gclc using an anti-Gclc antibody (1∶2000; Abnova). All data were normalized to the level of GAPDH and compared with age-matched wild type mice.

### Lens Protein Enzymatic Digestion for Advanced Glycation Endproduct Analysis

For AGE analysis by LC/MS, 1 mg of lens protein extract was enzymatically digested in Chelex treated phosphate buffer with sequential additions of peptidase (Sigma P7500), protease K, pronase and aminopeptidase M (Roche, IN) as described earlier [12]. Corresponding enzyme blanks were incubated without added protein as a background control. Protein concentration was determined using the ninhydrin assay, as described earlier [12].

### AGEs Determination by LC-MS/MS

Amounts of carboxymethyl-lysine (CML) and carboxyethyl-lysine (CEL), fructose-lysine (FL), methionine sulfoxide (MetSOX), glyoxal immidazolone-1 (G-H1) and methylglyoxal immidazolone-1 (MG-H1) in enzymatically digested samples were measured by electronspray positive ionization-mass spectrometric multiple reaction monitoring (ESI+MRM) with a LC-MS/MS system composed of a 2690 Separation module with a Quattro Ultima triple quadropole mass spectrometry detector (Water-Micromass, Manchester, U.K.) following the previously published procedure [40], [41]. Analytes released by self-digestion of proteases in assay blanks were subtracted from analytic estimates.

### Staining of Freshly Isolated Lenses with Hoechst 33342 and Dihydrorhodamine 123 (DHR)

Fresh isolated lenses were placed in chamber slides and stained with 7.5 µM dihydrorhodamine 123 (DHR) on ice for 45 min, followed by staining with 10 µM Hoechst 33342 for 15 min as described by Wolf et al [42]. The lens in M199 medium was then subjected to confocal image scanning.

### Image Analysis Using Confocal Microscope

A laser scanning confocal microscope (Carl Zeiss LSM510 META) was used for whole lens analysis upon vital staining with DNA fluorochrome Hoechst 33342 and ROS marker DHR. The excitation/emission at 405 nm/450 nm was used for Hoechst 3342 and 488 nm/550 nm was used for DHR image. Lens anterior was scanned using 10X objective, and 65 µm deep into the cortex and 0.8 µm layer Z-scan was performed. The image was analyzed with LSM images analysis software from Zeiss.

### Statistical Analysis

All values are expressed as means ± S.D. Statistical significance of difference in mean values was assessed by analysis of variance or Student’s *t* test. Only *p* values <0.05 were considered statistically significant.
